# Supplementary material for: Cancer-associated fibroblast classification in single-cell and spatial proteomics data
Source: Nat Commun. 2023 Jul 18;14:4294. doi: 10.1038/s41467-023-39762-1 (PMC10354071; doi:10.1038/s41467-023-39762-1)
Supplement: Supplementary file 1 — Supplementary Information [file 41467_2023_39762_MOESM1_ESM.pdf]

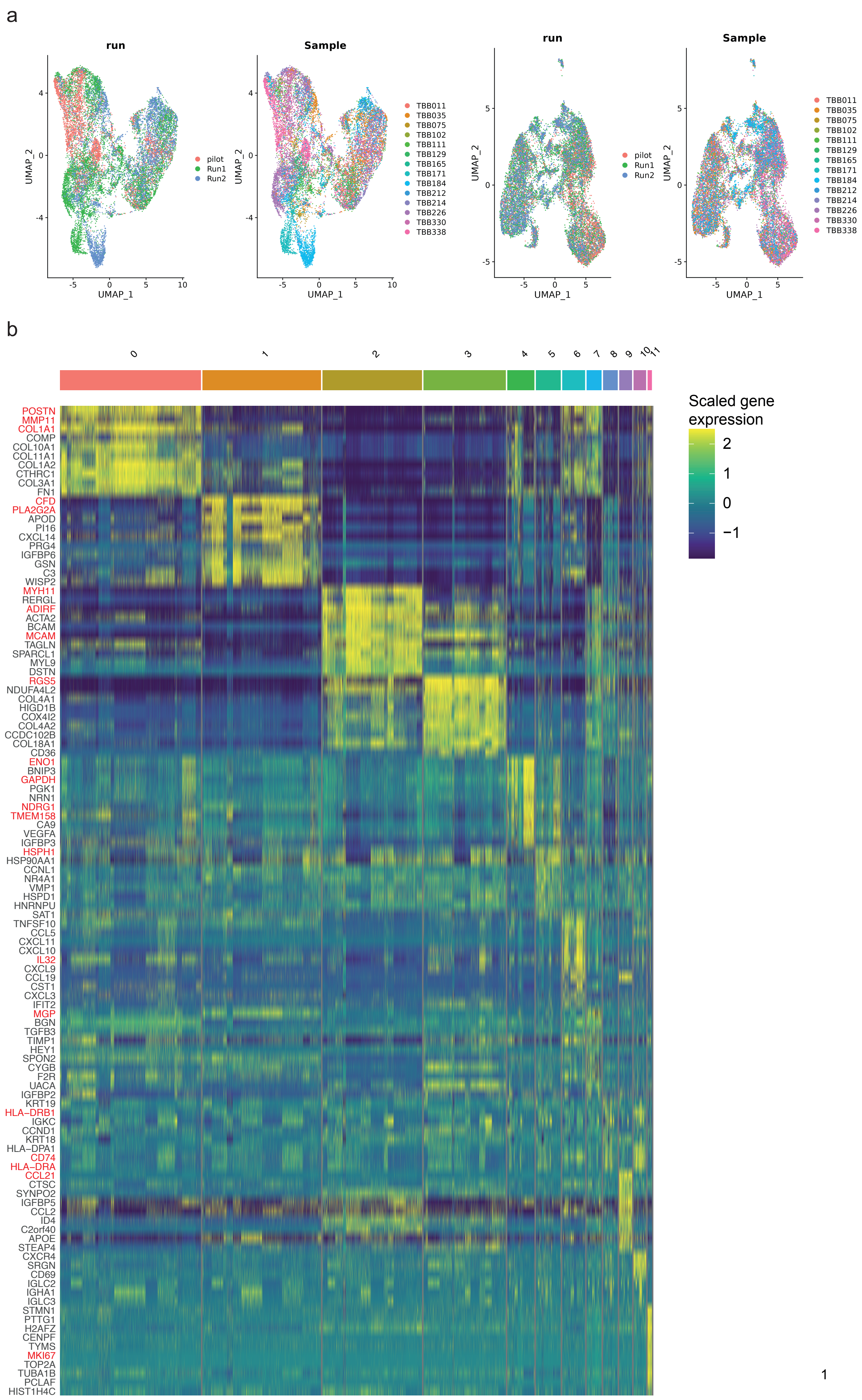
a
run
Sample
UMAP_1
UMAP_2
pilot
Run1
Run2
TBB011
TBB035
TBB075
TBB102
TBB111
TBB129
TBB165
TBB171
TBB184
TBB212
TBB214
TBB226
TBB330
TBB338
b
0 1 2 3 4 5 6 7 8 9 10 11
Scaled gene expression
2
1
0
−1
POSTN
MMP11
COL1A1
COMP
COL10A1
COL11A1
COL1A2
CTHRC1
COL3A1
FN1
CFD
PLA2G2A
APOD
PI16
CXCL14
PRG4
IGFBP6
GSN
C3
WISP2
MYH11
RERGL
ADIRF
ACTA2
BCAM
MCAM
TAGLN
SPARCL1
MYL9
DSTN
RGS5
NDUFA4L2
COL4A1
HIGD1B
COX4I2
COL4A2
CCDC102B
COL18A1
CD36
ENO1
BNIP3
GAPDH
PGK1
NRN1
NDRG1
TMEM158
CA9
VEGFA
IGFBP3
HSPH1
HSP90AA1
CCNL1
NR4A1
VMP1
HSPD1
HNRNPU
SAT1
TNFSF10
CCL5
CXCL11
CXCL10
IL32
CXCL9
CCL19
CST1
CXCL3
IFIT2
MGP
BGN
TGFB3
TIMP1
HEY1
SPON2
CYGB
F2R
UACA
IGFBP2
KRT19
HLA−DRB1
IGKC
CCND1
KRT18
HLA−DPA1
CD74
HLA−DRA
CCL21
CTSC
SYNPO2
IGFBP5
CCL2
ID4
C2orf40
APOE
STEAP4
CXCR4
SRGN
CD69
IGLC2
IGHA1
IGLC3
STMN1
PTTG1
H2AFZ
CENPF
TYMS
MKI67
TOP2A
TUBA1B
PCLAF
HIST1H4C

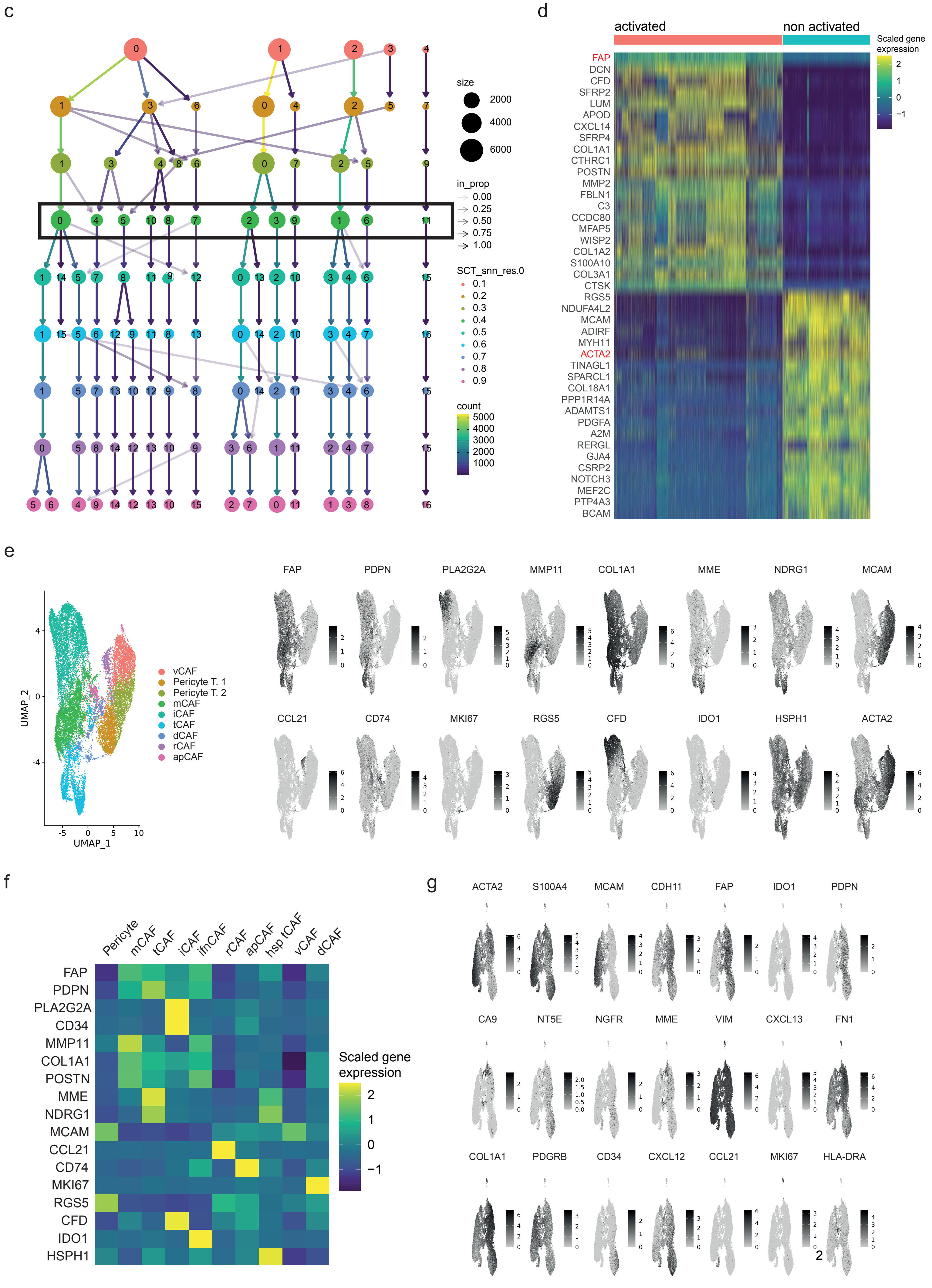

c
d
activated
non activated
Scaled gene expression
size
2000
4000
6000
in_prop
0.00
0.25
0.50
0.75
1.00
SCT_snn_res.0
0.1
0.2
0.3
0.4
0.5
0.6
0.7
0.8
0.9
count
5000
4000
3000
2000
1000
FAP
DCN
CFD
SFRP2
LUM
APOD
CXCL14
SFRP4
COL1A1
CTHRC1
POSTN
MMP2
FBLN1
C3
CCDC80
MFAP5
WISP2
COL1A2
S100A10
COL3A1
CTSK
RGS5
NDUFA4L2
MCAM
ADIRF
MYH11
ACTA2
TINAGL1
SPARCL1
COL18A1
PPP1R14A
ADAMTS1
PDGFA
A2M
RERGL
GJA4
CSRP2
NOTCH3
MEF2C
PTP4A3
BCAM
e
UMAP_2
UMAP_1
vCAF
Pericyte T. 1
Pericyte T. 2
mCAF
iCAF
tCAF
dCAF
rCAF
apCAF
FAP
PDPN
PLA2G2A
MMP11
COL1A1
MME
NDRG1
MCAM
CCL21
CD74
MKI67
RGS5
CFD
IDO1
HSPH1
ACTA2
f
Pericyte
mCAF
tCAF
iCAF
ifnCAF
rCAF
apCAF
hsp tCAF
vCAF
dCAF
FAP
PDPN
PLA2G2A
CD34
MMP11
COL1A1
POSTN
MME
NDRG1
MCAM
CCL21
CD74
MKI67
RGS5
CFD
IDO1
HSPH1
Scaled gene expression
g
ACTA2
S100A4
MCAM
CDH11
FAP
IDO1
PDPN
CA9
NT5E
NGFR
MME
VIM
CXCL13
FN1
COL1A1
PDGRB
CD34
CXCL12
CCL21
MKI67
HLA-DRA

Supplementary Figure 1 – scRNA-seq data of breast cancer

a) UMAPs of the uncorrected raw data (left two plots) and the batch corrected data (right two plots), coloured by run and sample ID.
b) Heatmap showing the top 10 differentially expressed genes for each cluster at the resolution of 0.4.
c) Clustree showing hierarchical clustering for breast cancer fibroblasts.
d) Heatmap of all cells showing the classification as activated and non-activated and their respective differentially expressed genes.
e) UMAP of CAF type assignment from raw, uncorrected scRNA-Seq data. The feature plots show expression level of our defined CAF selected marker genes.
f) Heatmap showing the average marker gene expression of all defined marker genes for clusters detected when clustering all stromal cells using only the subset of defined marker genes.
g) Feature plot showing expression of all CAF markers selected for IMC as detected in the breast cancer cohort.

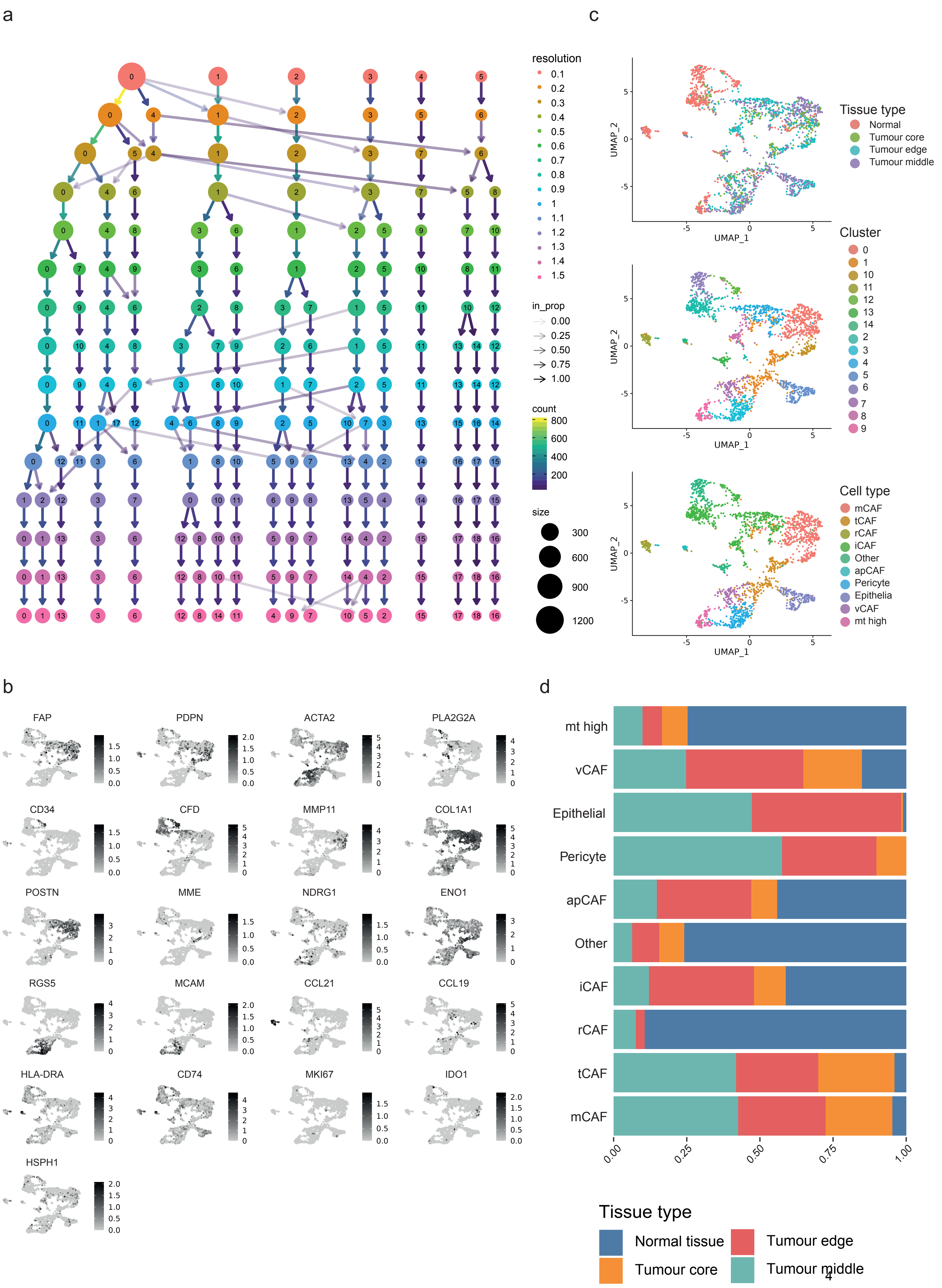
a
resolution
0.1
0.2
0.3
0.4
0.5
0.6
0.7
0.8
0.9
1
1.1
1.2
1.3
1.4
1.5
in_prop
0.00
0.25
0.50
0.75
1.00
count
800
600
400
200
size
300
600
900
1200
b
FAP
PDPN
ACTA2
PLA2G2A
CD34
CFD
MMP11
COL1A1
POSTN
MME
NDRG1
ENO1
RGS5
MCAM
CCL21
CCL19
HLA-DRA
CD74
MKI67
IDO1
HSPH1
c
UMAP_1
UMAP_2
Tissue type
Normal
Tumour core
Tumour edge
Tumour middle
Cluster
0
1
10
11
12
13
14
2
3
4
5
6
7
8
9
Cell type
mCAF
tCAF
rCAF
iCAF
Other
apCAF
Pericyte
Epithelia
vCAF
mt high
d
mt high
vCAF
Epithelial
Pericyte
apCAF
Other
iCAF
rCAF
tCAF
mCAF
0.00
0.25
0.50
0.75
1.00
Tissue type
Normal tissue
Tumour edge
Tumour core
Tumour middle

Supplementary Figure 2 – Analysis of the lung cancer scRNA-seq data

a) Clustree showing the hierarchical clustering of all lung cancer stromal cells.
b) Feature plot showing the marker expression of all marker genes for all cells in this dataset.
c) UMAPs coloured by (top to bottom) tissue sites sequenced in the original study (normal tissue, tumour core, tumour edge, tumour middle), clusters obtained at clustering resolution of 0.8, and the final cell type assignments of mCAFs, tCAFs, rCAFs, iCAFs, other, apCAFs, pericytes, epithelial cells, vCAFs, and mitochondrial-high cells (mt high).
d) Proportions of tissue site distribution of all defined cell types in this dataset.

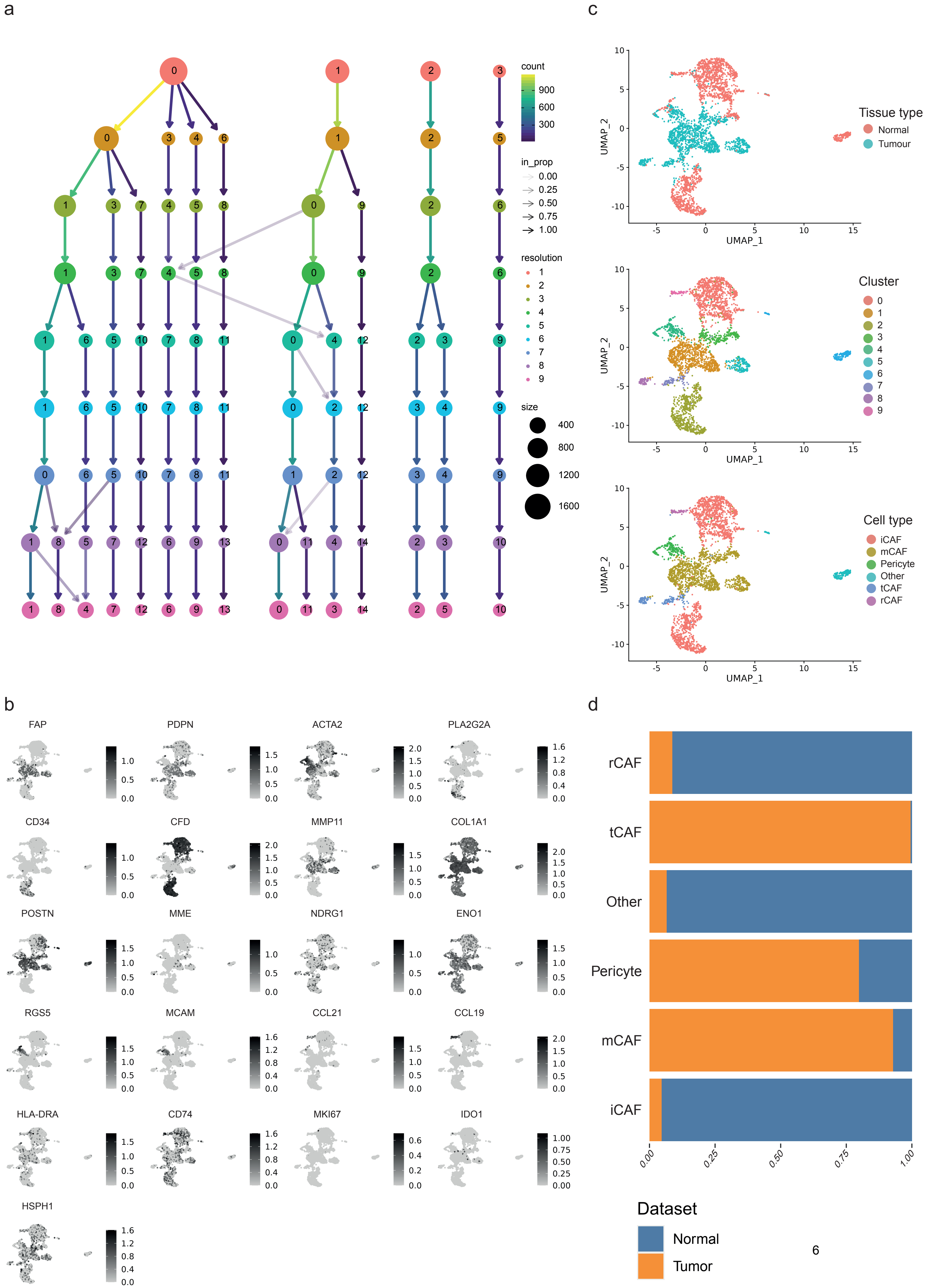
a
b
c
d
count
900
600
300
in_prop
0.00
0.25
0.50
0.75
1.00
resolution
size
400
800
1200
1600
FAP
PDPN
ACTA2
PLA2G2A
CD34
CFD
MMP11
COL1A1
POSTN
MME
NDRG1
ENO1
RGS5
MCAM
CCL21
CCL19
HLA-DRA
CD74
MKI67
IDO1
HSPH1
Tissue type
Normal
Tumour
Cluster
Cell type
iCAF
mCAF
Pericyte
Other
tCAF
rCAF
UMAP_1
UMAP_2
rCAF
tCAF
Other
Pericyte
mCAF
iCAF
Dataset
Normal
Tumor

Supplementary Figure 3 – Analysis of scRNA-seq data from colon cancer

a) Clustree showing the hierarchical clustering of all colon cancer stromal cells.
b) Feature plot of all colon cancer CAFs showing the expression of defined marker genes.
c) UMAPs coloured by (top to bottom) tissue sites sequenced in the original study (normal tissue, tumour tissue), clusters obtained at clustering resolution of 0.3, and final cell type assignments of iCAFs, mCAFs, pericytes, other, tCAFs, and rCAFs.
d) Cell type distribution between tumour and healthy tissue origin for all cell types defined in this dataset.

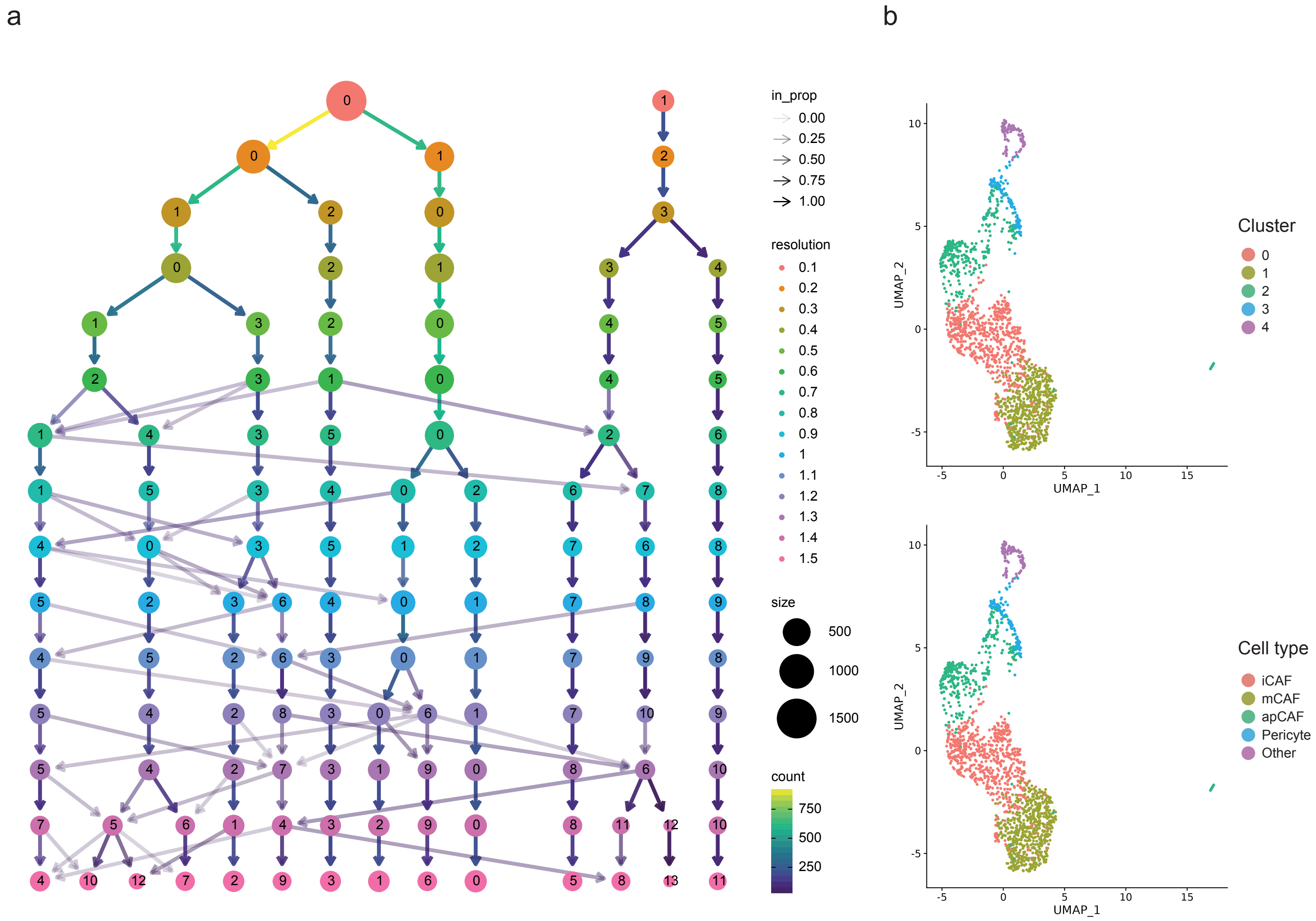
a
b
in_prop
0.00
0.25
0.50
0.75
1.00
resolution
0.1
0.2
0.3
0.4
0.5
0.6
0.7
0.8
0.9
1
1.1
1.2
1.3
1.4
1.5
size
500
1000
1500
count
750
500
250
UMAP_2
UMAP_1
Cluster
0
1
2
3
4
Cell type
iCAF
mCAF
apCAF
Pericyte
Other

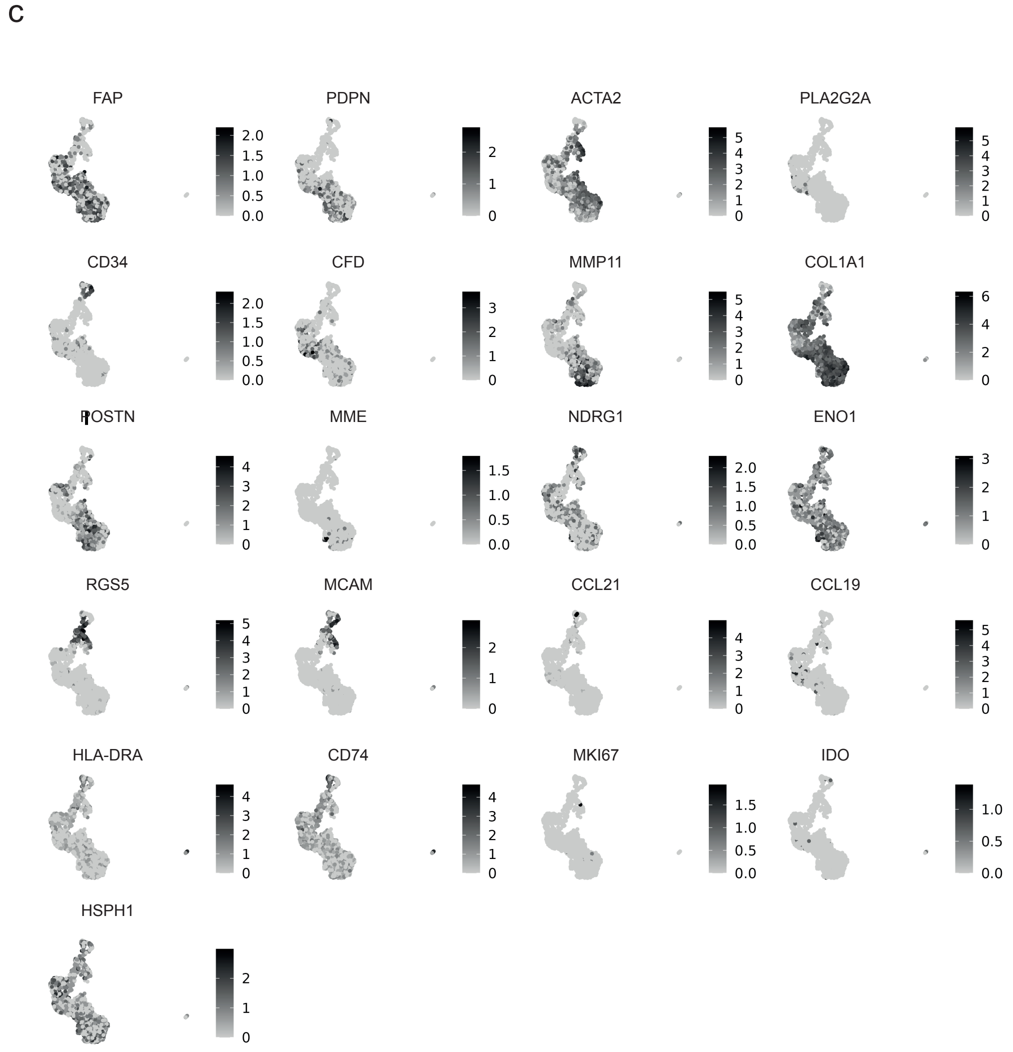
c
FAP
PDPN
ACTA2
PLA2G2A
CD34
CFD
MMP11
COL1A1
POSTN
MME
NDRG1
ENO1
RGS5
MCAM
CCL21
CCL19
HLA-DRA
CD74
MKI67
IDO
HSPH1

Supplementary Figure 4 – Individual analysis of the small PDAC dataset.

a) Hierarchical clustering tree (using the clustree package) showing the clustering results of the small PDAC dataset at clustering steps.

b) UMAPs coloured by the chosen clustering resolution of 0.4 and the resulting 5 different clusters as well as the final cluster assignment (iCAFs, mCAFs, apCAFs, pericytes and other).

c) Feature plot showing the expression of all previously identified marker genes on the UMAP.

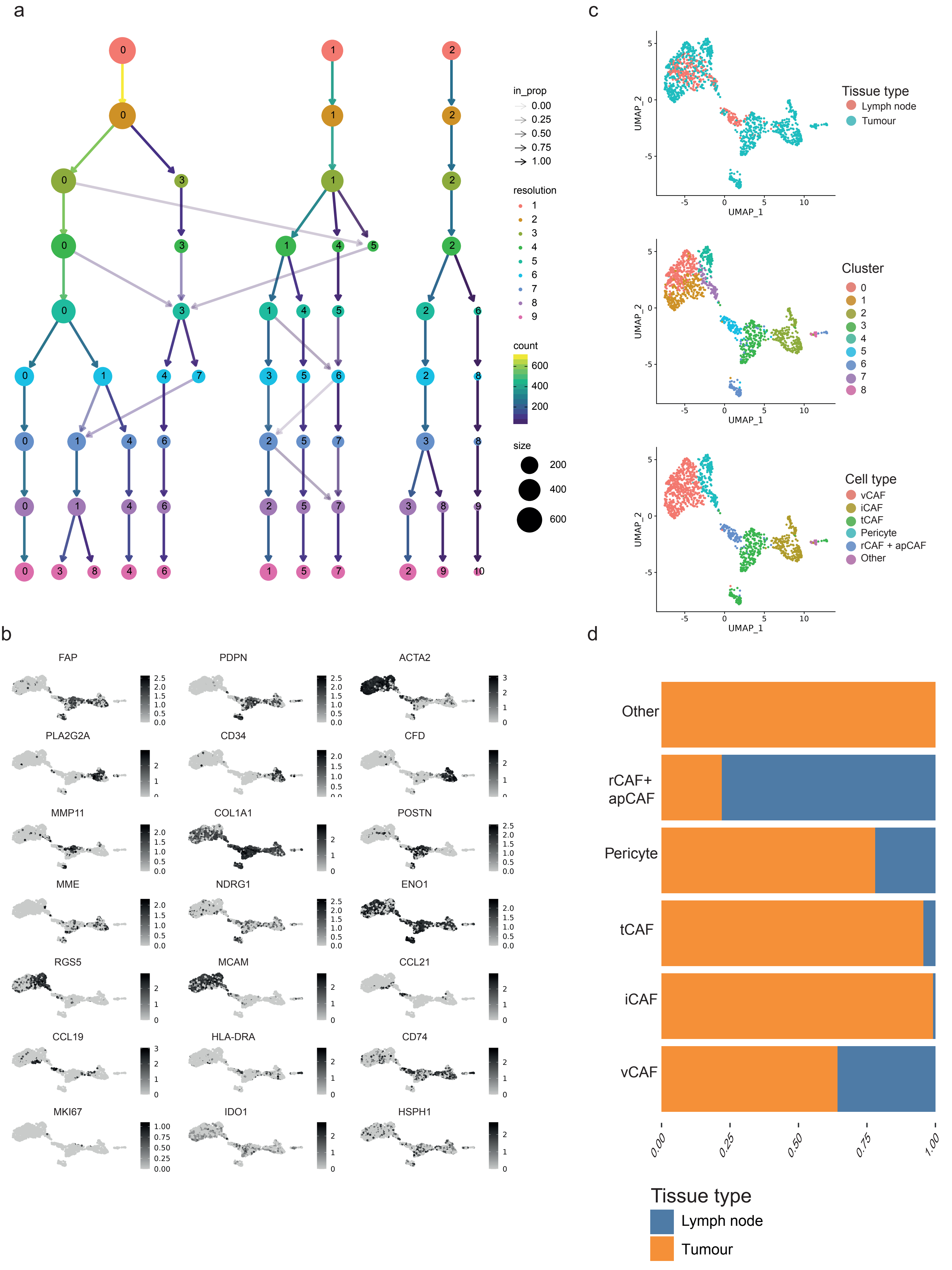
a
in_prop
0.00
0.25
0.50
0.75
1.00
resolution
count
600
400
200
size
200
400
600
b
FAP
PDPN
ACTA2
PLA2G2A
CD34
CFD
MMP11
COL1A1
POSTN
MME
NDRG1
ENO1
RGS5
MCAM
CCL21
CCL19
HLA-DRA
CD74
MKI67
IDO1
HSPH1
c
UMAP_1
UMAP_2
Tissue type
Lymph node
Tumour
Cluster
Cell type
vCAF
iCAF
tCAF
Pericyte
rCAF + apCAF
Other
d
Other
rCAF+ apCAF
Pericyte
tCAF
iCAF
vCAF
0.00
0.25
0.50
0.75
1.00
Tissue type
Lymph node
Tumour

Supplementary Figure 5 - Analysis of scRNA-seq data from head and neck squamous cell carcinoma

a) Clustree showing clustering hierarchy of HNSCC CAFs. Feature plot showing the marker expression of all marker genes for all cells in this dataset.
b) Feature plot showing the expression of all previously identified marker genes on the UMAP.
c) UMAPs coloured by (top to bottom) tissue sites sequenced in the original study (metastatic lymph node, primary tumour tissue), clusters obtained at clustering resolution of 0.6, and the final cell type assignments of vCAFs, iCAFs, tCAFs, pericytes, apCAFs + rCAFs, and other cells.
d) Cell type distribution between tumour and metastatic lymph node tissue origin for all cell types defined in this dataset.

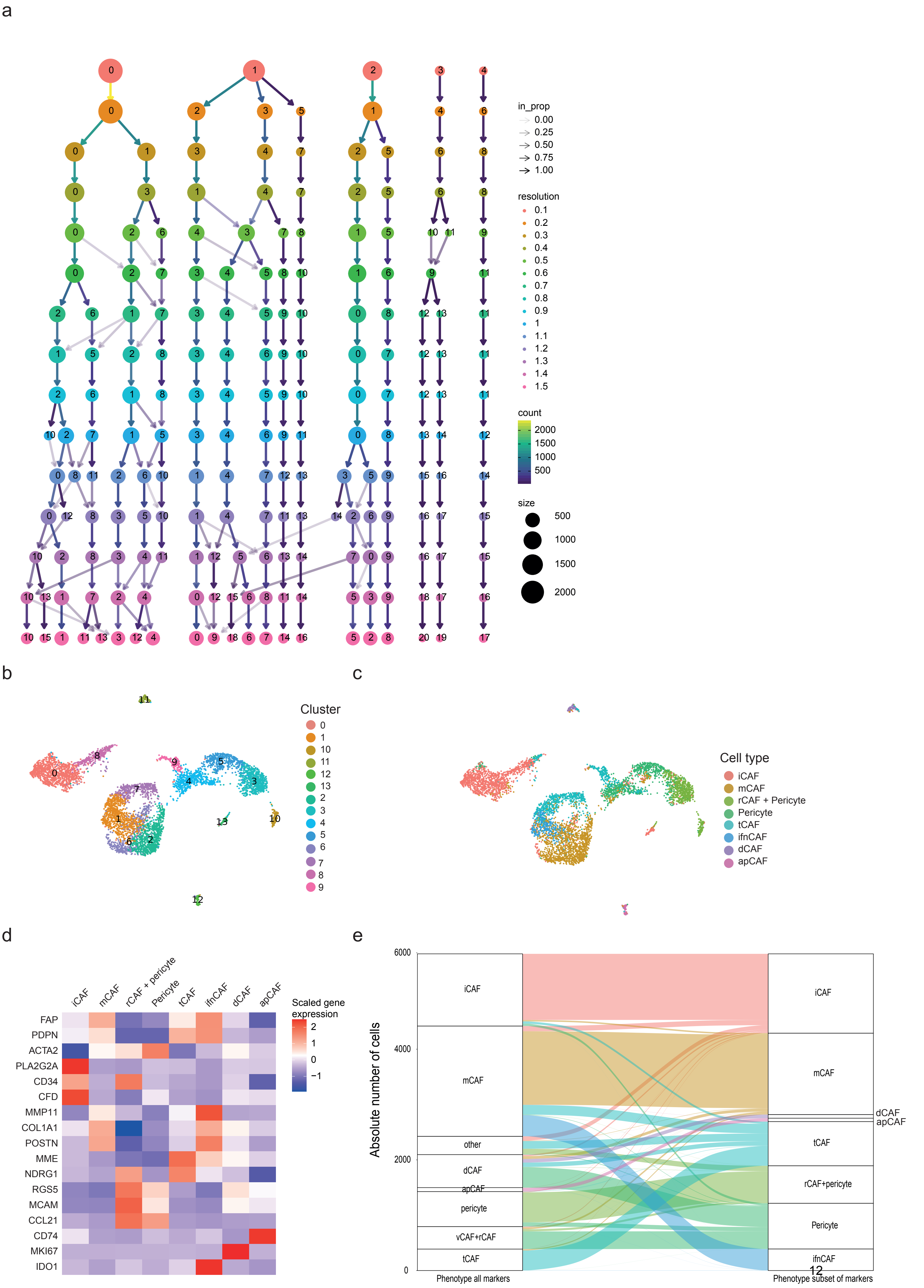
a
in_prop
0.00
0.25
0.50
0.75
1.00
resolution
0.1
0.2
0.3
0.4
0.5
0.6
0.7
0.8
0.9
1
1.1
1.2
1.3
1.4
1.5
count
2000
1500
1000
500
size
500
1000
1500
2000
b
Cluster
0
1
10
11
12
13
2
3
4
5
6
7
8
9
c
Cell type
iCAF
mCAF
rCAF + Pericyte
Pericyte
tCAF
ifnCAF
dCAF
apCAF
d
iCAF
mCAF
rCAF + pericyte
Pericyte
tCAF
ifnCAF
dCAF
apCAF
Scaled gene expression
2
1
0
−1
FAP
PDPN
ACTA2
PLA2G2A
CD34
CFD
MMP11
COL1A1
POSTN
MME
NDRG1
RGS5
MCAM
CCL21
CD74
MKI67
IDO1
e
Absolute number of cells
6000
4000
2000
0
iCAF
mCAF
other
dCAF
apCAF
pericyte
vCAF+rCAF
tCAF
Phenotype all markers
iCAF
mCAF
dCAF
apCAF
tCAF
rCAF+pericyte
Pericyte
ifnCAF
Phenotype subset of markers

Supplementary Figure 6 - Analysis of the integrated scRNA-seq datasets

a) Clustree showing the hierarchical clustering results of the integrated dataset.
b) Clustering of the integrated dataset at 0.7 chosen for the final cell type assignment.
c) UMAP coloured by CAF types recovered when clustering the integrated dataset using only the subset of defined marker genes.
d) Heatmap showing the average expression level of defined marker genes for clusters defined after clustering the dataset using only the defined marker genes.
e) Sankey plot showing the cell affiliations when clustering using (left) all genetic features of the integrated dataset and (right) only the subset of defined marker genes.

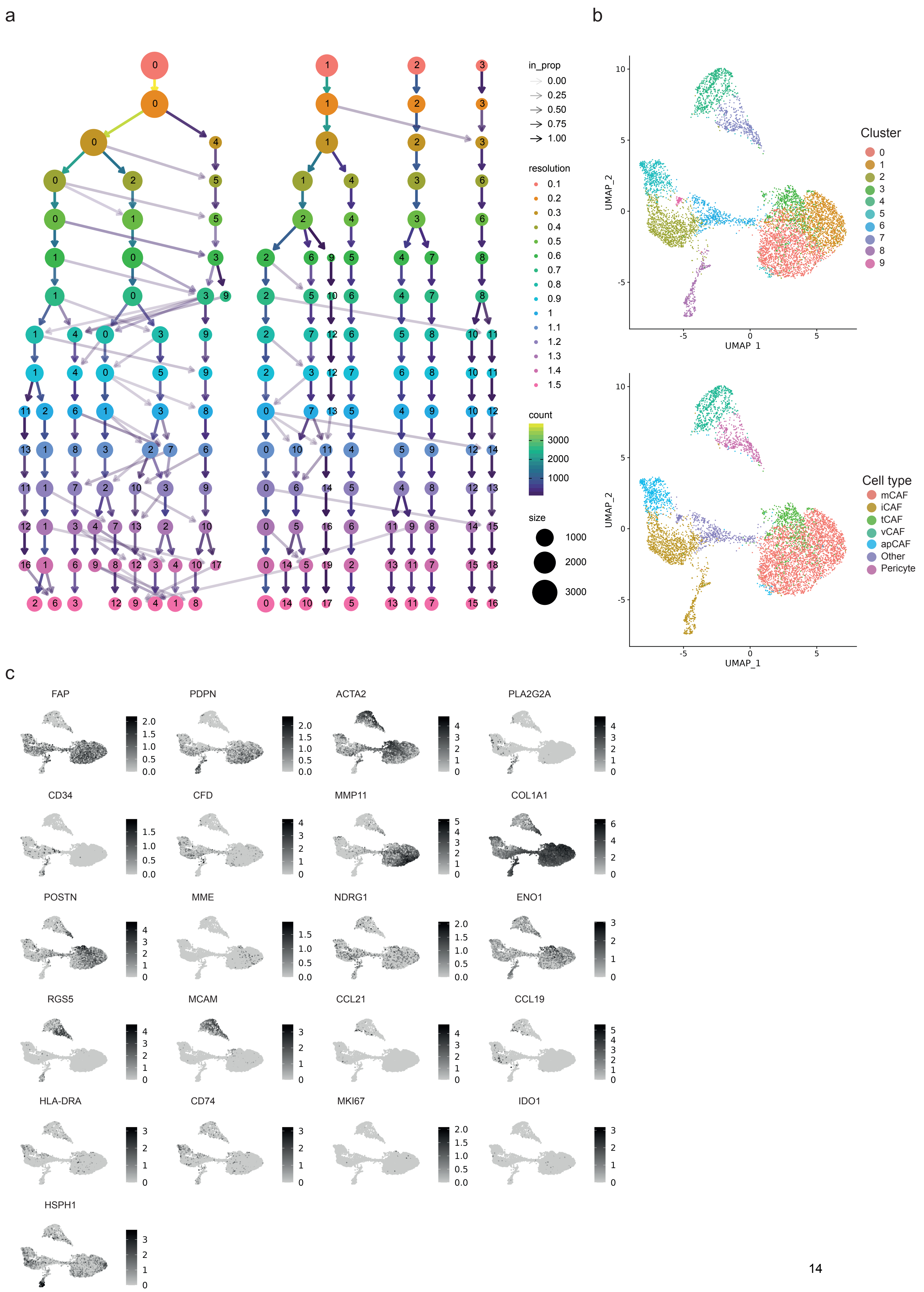
a
b
c
in_prop
0.00
0.25
0.50
0.75
1.00
resolution
0.1
0.2
0.3
0.4
0.5
0.6
0.7
0.8
0.9
1
1.1
1.2
1.3
1.4
1.5
count
3000
2000
1000
size
1000
2000
3000
UMAP_2
UMAP_1
Cluster
Cell type
mCAF
iCAF
tCAF
vCAF
apCAF
Other
Pericyte
FAP
PDPN
ACTA2
PLA2G2A
CD34
CFD
MMP11
COL1A1
POSTN
MME
NDRG1
ENO1
RGS5
MCAM
CCL21
CCL19
HLA-DRA
CD74
MKI67
IDO1
HSPH1

Supplementary Figure 7 – Individual analysis of a larger PDAC dataset

a) Hierarchical clustering tree (using the clustree package) showing the clustering results of the big PDAC dataset.
b) UMAPs coloured by the chosen clustering resolution of 0.6 and the resulting 10 different clusters as well as the final cluster assignment.
c) Feature plot showing the expression of all previously identified marker genes on the UMAP.

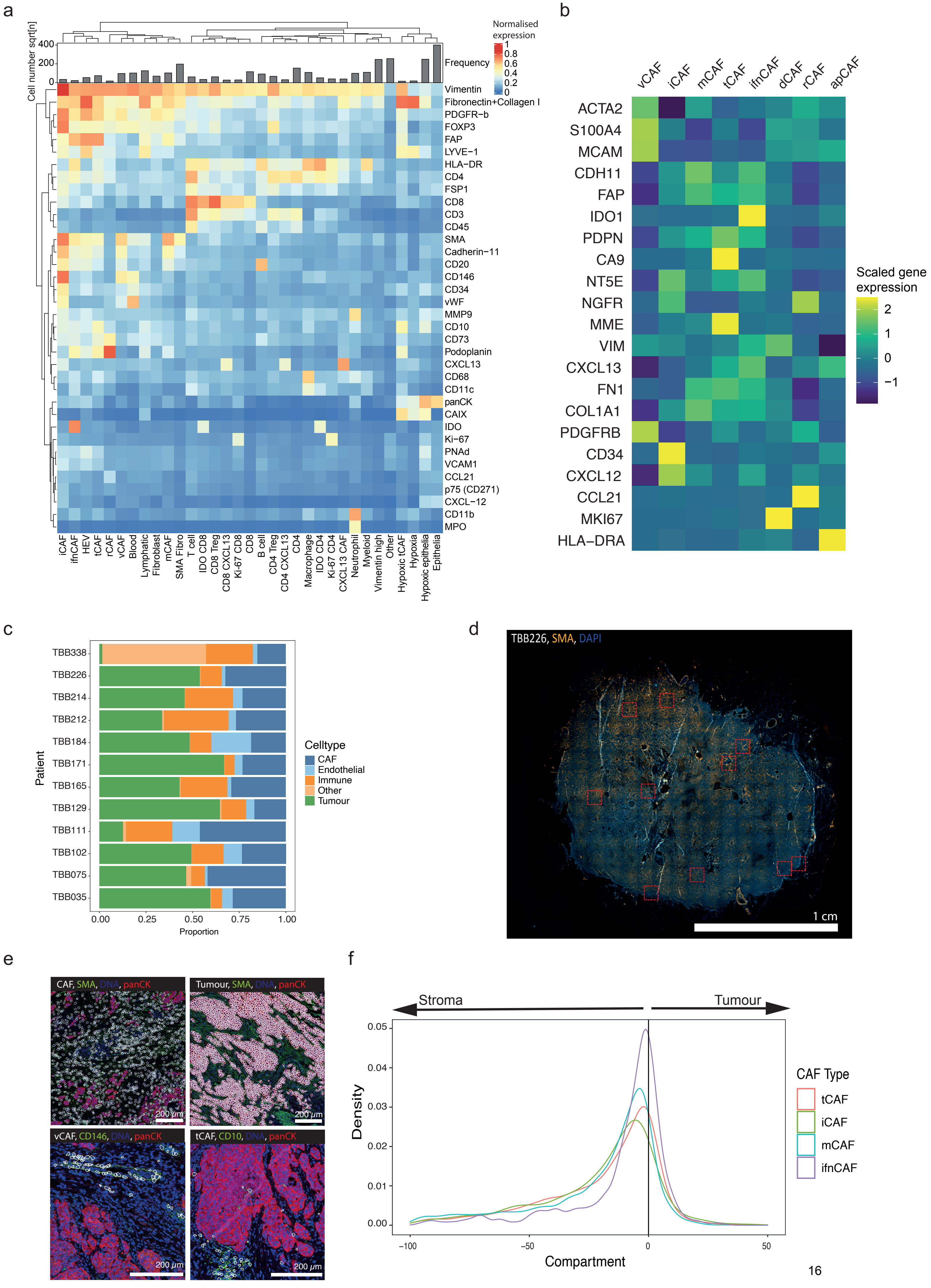

a
Normalised expression
Cell number sqrt[n]
Frequency
Vimentin
Fibronectin+Collagen I
PDGFR−b
FOXP3
FAP
LYVE−1
HLA−DR
CD4
FSP1
CD8
CD3
CD45
SMA
Cadherin−11
CD20
CD146
CD34
vWF
MMP9
CD10
CD73
Podoplanin
CXCL13
CD68
CD11c
panCK
CAIX
IDO
Ki−67
PNAd
VCAM1
CCL21
p75 (CD271)
CXCL−12
CD11b
MPO
iCAF
ifnCAF
HEV
tCAF
rCAF
vCAF
Blood
Lymphatic
Fibroblast
mCAF
SMA Fibro
T cell
IDO CD8
CD8 Treg
CD8 CXCL13
Ki-67 CD8
CD8
B cell
CD4 Treg
CD4 CXCL13
CD4
Macrophage
IDO CD4
Ki-67 CD4
CXCL13 CAF
Neutrophil
Myeloid
Vimentin high
Other
Hypoxic tCAF
Hypoxia
Hypoxic epithelia
Epithelia
b
vCAF
iCAF
mCAF
tCAF
ifnCAF
dCAF
rCAF
apCAF
ACTA2
S100A4
MCAM
CDH11
FAP
IDO1
PDPN
CA9
NT5E
NGFR
MME
VIM
CXCL13
FN1
COL1A1
PDGFRB
CD34
CXCL12
CCL21
MKI67
HLA−DRA
Scaled gene expression
c
Patient
TBB338
TBB226
TBB214
TBB212
TBB184
TBB171
TBB165
TBB129
TBB111
TBB102
TBB075
TBB035
Proportion
Celltype
CAF
Endothelial
Immune
Other
Tumour
d
TBB226, SMA, DAPI
1 cm
e
CAF, SMA, DNA, panCK
Tumour, SMA, DNA, panCK
vCAF, CD146, DNA, panCK
tCAF, CD10, DNA, panCK
200 μm
f
Stroma
Tumour
Density
Compartment
CAF Type
tCAF
iCAF
mCAF
ifnCAF

Supplementary Figure 8 – IMC analysis of matched breast cancer tissue samples

a) Heatmap showing the average marker expression of all markers assessed in this study for all assigned cell types. Bar chart shows the number of cells per cluster.
b) Average marker expression per CAF cluster in the breast cancer scRNA-seq data, when clustering based on the genes selected for IMC analysis.
c) Bar chart showing the proportional distribution of CAFs, endothelial, immune, other cells, and tumour cells per patient as measured with IMC.
d) Whole slide immunofluorescence image of sample TBB showing the region selection for IMC based on the stroma distribution. All images were acquired once.
e) Images showing the expression of Iridium, SMA, panCK, CD146 and CD10 for a selection of images together with an overlay of cell masks showing the indicated cell types (all CAFs, tumour cells, vCAFs or tCAFs) in the image. The images showing vCAFs and tCAFs are zoomed-in areas to allow better visualisation of the CAF types in the tissue. Scale bars, 200 µm. All images were acquired once.
f) Density plots showing the distance of $CD34^+$ iCAFs, tCAFs, mCAFs, and ifnCAFs to the tumour stroma border (stroma to the left, tumour on the right).

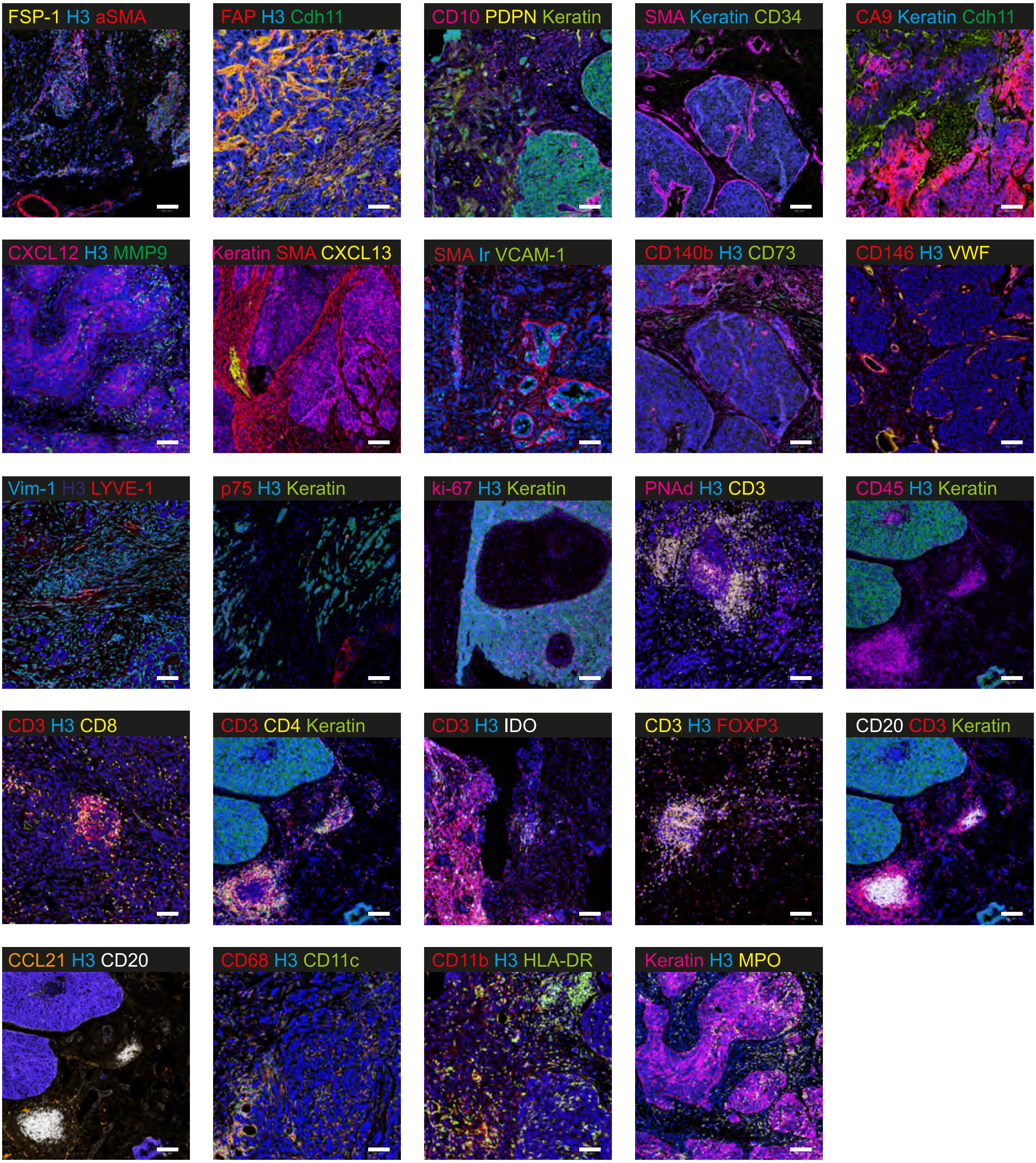
FSP-1 H3 aSMA
FAP H3 Cdh11
CD10 PDPN Keratin
SMA Keratin CD34
CA9 Keratin Cdh11
CXCL12 H3 MMP9
Keratin SMA CXCL13
SMA Ir VCAM-1
CD140b H3 CD73
CD146 H3 VWF
Vim-1 H3 LYVE-1
p75 H3 Keratin
ki-67 H3 Keratin
PNAd H3 CD3
CD45 H3 Keratin
CD3 H3 CD8
CD3 CD4 Keratin
CD3 H3 IDO
CD3 H3 FOXP3
CD20 CD3 Keratin
CCL21 H3 CD20
CD68 H3 CD11c
CD11b H3 HLA-DR
Keratin H3 MPO

Supplementary Figure 9 – All measured IMC marker channels shown once

All markers measured in IMC shown at least once as detected in tissues used in this study. Scale bars, 200 μm. All images were acquired once.

a

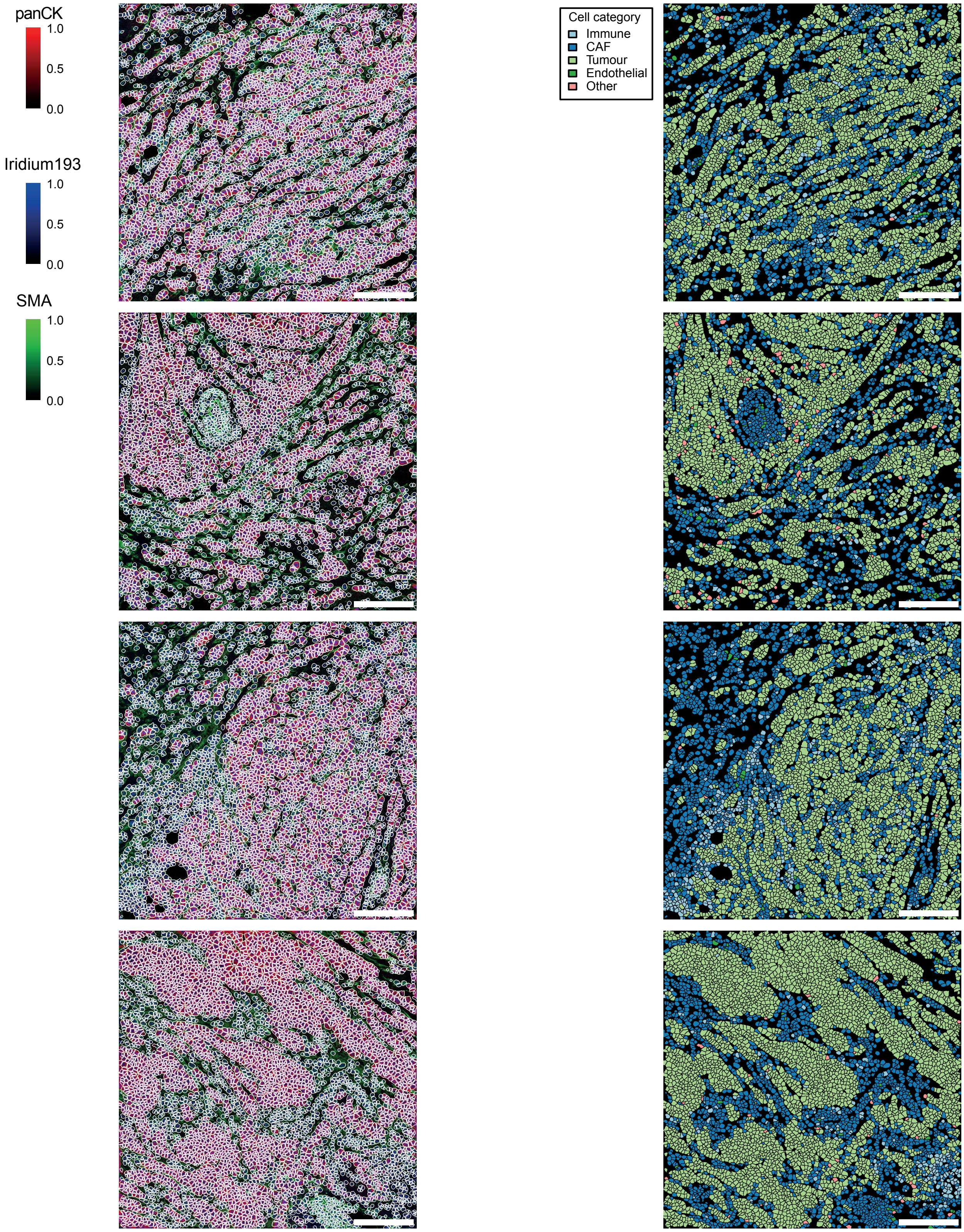

panCK
1.0
0.5
0.0
Iridium193
1.0
0.5
0.0
SMA
1.0
0.5
0.0
Cell category
Immune
CAF
Tumour
Endothelial
Other

b

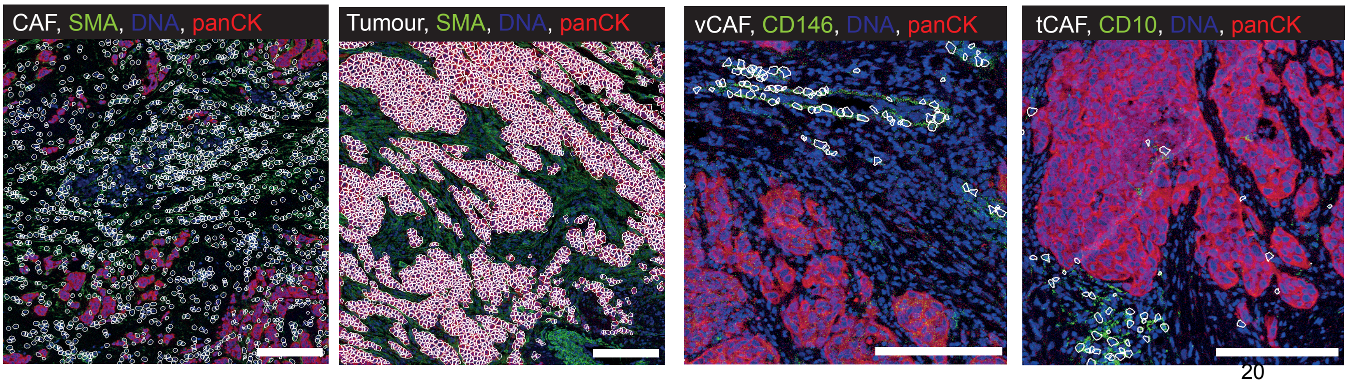

CAF, SMA, DNA, panCK
Tumour, SMA, DNA, panCK
vCAF, CD146, DNA, panCK
tCAF, CD10, DNA, panCK

Supplementary Figure 10 – IMC single-cell segmentation mask overlays

a) Raw pixel images (left) showing pan CK in red, SMA in green, DNA in blue with overlaying cell segmentation masks (cells in white). Pseudo-coloured segmentation masks (right) for all cell categories (tumour, CAF, endothelial, immune, other) defined in our IMC study. Scale bars, 200 μm. All images were acquired once.

b) Raw pixel images showing expression of the indicated markers, with cell segmentation masks (white) also shown for CAFs, tumour cells, vCAFs and tCAFs with markers showing SMA in green (first two images), CD146 in green (third image) and CD10 in green (fourth image), DNA (Iridium) in blue and panCK in red. Scale bars, 200 μm. All images were acquired once.

Metal Tag	Target	Antibody Clone	Concentration	Lot	Provider	Catalogue Number	RRID
Y89	Myeloperoxidase	Polyclonal	4 ug/mL	2003045	Dako	A0398	AB_2335676
In113	FSP1 / S100A4	Polyclonal	6 ug/mL	3126953	Millipore	136784	2
In115	SMA	1A4	2 ug/mL	2183900	E-Bioscience	14-9760-82	AB_2572996
Pr141	Histone H3	D1H2	1 ug/mL	15	CST	4499BF	7
Nd142	CD11b	SP330	5 ug/mL	GR3258740-1	Abcam	ab241408	AB_2889379
Nd143	HLA-DR	TAL 1B5	1 ug/mL	GR3222279-4	Abcam	ab20181	AB_445401
Nd144	CD146	Polyclonal	2 ug/mL	ECL0319041	R&D Systems	AF932	AB_355721
Nd145	Cadherin-11	283416	5 ug/mL	VLT0219091	R&D Systems	MAB1790	AB_2076970
Nd146	fap	Polyclonal	5 ug/mL	ZKW0315081	R&D Systems	AF3715	AB_2102369
Sm147	CD11b	M1/70	5 ug/mL	B186484	Biolegend	101202	AB_312785
Nd148	VCAM1	EPR5047	5 ug/mL	GR3255420-4	Abcam	ab215380	AB_2894839
Sm149	CD20	L26	4 ug/mL	2059976	E-Bioscience	14-0202-82	AB_10734340
Nd150	CD68	KP1	4 ug/mL	2130291	E-Bioscience	14-0688-82	AB_11151139
Eu151	Indoleamine 2- 3-dioxygenase	SP260	5 ug/mL	GR3259345-2	Abcam	ab245737	AB_2894840
Sm152	CD3	Polyclonal	6 ug/mL	GR3229164-6	Dako	A0452	AB_2335677
Eu153	Podoplanin	NC-08	5 ug/mL	B260834	Biolegend	337002	AB_1595511
Sm154	CD11c	D3V1E	5 ug/mL	2	CST	45581BF	AB_2799286
Gd155	Carbonic Anhydrase IX	Polyclonal	4 ug/mL	VNQ0319011	R&D Systems	AF2188	AB_416562
Gd156	CD73	D7F9A	4 ug/mL	2	CST	13160BF	AB_2716625
Gd158	MMP9	D6O3H	5 ug/mL	4	CST	13667BF	AB_2798289
Tb159	p75 / CD271	Polyclonal	10 ug/mL	ANT007AN0702	alomone labs	ANT-007	AB_2039968
Dy161	CD10	E5P7S	5 ug/mL	2	CST	65534	AB_2894842
Dy162	Vimentin	EPR3776	1 ug/mL	GR286525-2	Abcam	ab193555	AB_2814713
Dy163	FOXP3	236A/E7	5 ug/mL	2129676	E-Bioscience	14-4777-82	AB_467556
Dy164	CXCL13	Polyclonal	4 ug/mL	BAS0317101	R&D Systems	AF801	AB_355613
Ho165	PNAd	MECA-79	4 ug/mL	B257139	Biolegend	120802	AB_493555
Er166	CD8a	C8/144B	4 ug/mL	2132595	E-Bioscience	14-0085-82	AB_11150240
Er167	Fibronectin	10/Fibronectin	3 ug/mL	6251888	Bection Dickinson	610078	AB_397486
Er167	Collagen I	Polyclonal	4 ug/mL	GR3253239-3	Abcam	ab34710	AB_731684
Er168	LYVE-1	Polyclonal	7 ug/mL	KPY0119052	R&D Systems	AF2089	AB_355144
Tm169	CD140b	Y92	6 ug/mL	GR296584-4	Abcam	ab215978	AB_2894841
Er170	CD34	EP373Y	10 ug/mL	GR3271518-1	Abcam	ab198395	AB_2889381
Yb171	CD4	Polyclonal	5 ug/mL	YS0718031	R&D Systems	AF-379-NA	AB_354469
Yb172	vWF	poly vwf	4 ug/mL	3322998	Millipore	AB7356	AB_92216
Yb172	CD31	EPR3094	4 ug/mL	GR3229164-6	Abcam	ab207090	AB_2889382
Yb173	CXCL12 / SDF-1	79018	5 ug/mL	JOJ0519031	R&D Systems	MAB350-100	AB_2088149
Yb174	CCL21 / 6Ckine	Polyclonal	6 ug/mL	AYJ218071	R&D Systems	AF366	AB_355327
Lu175	Keratin Epithelial	AE3	2 ug/mL	3255457	Millipore	MAB1611	AB_2134409
Lu175	pan Cytokeratin	AE1	2 ug/mL	3252910	Millipore	MAB1612	AB_2132794
Pt194	Ki-67	B56	5 ug/mL	8116751	Bection Dickinson	556003	AB_396287
Bi209	CD45	2B11	3 ug/mL	2003422	E-Bioscience	14-9457-82	AB_11063696
AF 555	Goat anti-mouse (H+L)	Polyclonal	1 : 500 dilution	2090527	Sigma Aldrich	A21422	AB_2535844

Supplementary Table 1 – Antibody panel and Research Resource Identifiers (RRID) validation

Table showing antibody panel, metal tags, antibody clone information and RRID validation information.
