# Supplementary material for: Cancer-associated fibroblast classification in single-cell and spatial proteomics data
Source: Nat Commun. 2023 Jul 18;14:4294. doi: 10.1038/s41467-023-39762-1 (PMC10354071; doi:10.1038/s41467-023-39762-1)
Supplement: Supplementary file 3 — Description of Additional Supplementary Files [file 41467_2023_39762_MOESM3_ESM.pdf]

Description of Additional Supplementary Files

Supplementary Data 1 – Clinical data and 10X run information Anonymised clinical data of all pa1ents used in the breast cancer dataset.

Supplementary Data 2 – Differential gene expression analysis of breast cancer clusters as seen at resolution 0.4 Table showing results of differen1al gene expression analysis of all clusters as seen at resolu1on 0.4 before merging clusters to phenotypes. The differen1al gene expression (MAST) analysis was done using two-sided and p values were adjusted by Bonferroni (p_val_adj).

Supplementary Data 3 – Differential gene expression analysis of all CAF and pericyte phenotypes Table showing results of differen1al gene expression analysis of all defined CAF and pericyte phenotypes. The differen1al gene expression (MAST) analysis was done using two-sided and p values were adjusted by Bonferroni (p_val_adj).

Supplementary Data 4 – Differential gene expression analysis comparing FAP+ and FAPCAFs. Table showing results of differen1al gene expression analysis of FAP+ versus FAP- cells. The differen1al gene expression (MAST) analysis was done using two-sided and p values were adjusted by Bonferroni (p_val_adj).

Supplementary Data 5 – Differential gene expression analysis of all clusters in the integrated dataset. Table showing results of differen1al gene expression analysis of all defined CAF and pericyte clusters in all datasets aKer dataset integra1on at the resolu1on of 0.7. The differen1al gene expression (MAST) analysis was done using two-sided and p values were adjusted by Bonferroni (p_val_adj).

Supplementary Data 6 – Differential gene expression analysis of all phenotypes in the integrated dataset, after merging of clusters. Table showing results of differen1al gene expression analysis of all defined CAF and pericyte phenotypes in all datasets aKer dataset integra1on. The differen1al gene expression (MAST) analysis was done using two-sided and p values were adjusted by Bonferroni (p_val_adj).

Supplementary Data 7 – Comparison of gene set enrichment analysis in breast cancer dataset and integrated dataset Table showing the top 20 hallmark pathways for each CAF type, for the breast cancer and integrated validation datasets. Overlapping pathways are highlighted in green, and defining pathways are highlighted in bold in the breast cancer data.
