# Supplementary material for: Cancer-associated fibroblast classification in single-cell and spatial proteomics data
Source: Nat Commun. 2023 Jul 18;14:4294. doi: 10.1038/s41467-023-39762-1 (PMC10354071; doi:10.1038/s41467-023-39762-1)
Supplement: Supplementary file 11 — Reporting Summary [file 41467_2023_39762_MOESM11_ESM.pdf]

nature portfolio

Corresponding author(s): Bernd Bodenmiller

Last updated by author(s): Jun 12, 2023

Reporting Summary

Nature Portfolio wishes to improve the reproducibility of the work that we publish. This form provides structure for consistency and transparency in reporting. For further information on Nature Portfolio policies, see our Editorial Policies and the Editorial Policy Checklist.

Statistics

For all statistical analyses, confirm that the following items are present in the figure legend, table legend, main text, or Methods section.

n/a	Confirmed	
☐	☒	The exact sample size (*n*) for each experimental group/condition, given as a discrete number and unit of measurement
☐	☒	A statement on whether measurements were taken from distinct samples or whether the same sample was measured repeatedly
☐	☒	The statistical test(s) used AND whether they are one- or two-sided<br>*Only common tests should be described solely by name; describe more complex techniques in the Methods section.*
☒	☐	A description of all covariates tested
☐	☒	A description of any assumptions or corrections, such as tests of normality and adjustment for multiple comparisons
☐	☒	A full description of the statistical parameters including central tendency (e.g. means) or other basic estimates (e.g. regression coefficient) AND variation (e.g. standard deviation) or associated estimates of uncertainty (e.g. confidence intervals)
☐	☒	For null hypothesis testing, the test statistic (e.g. *F*, *t*, *r*) with confidence intervals, effect sizes, degrees of freedom and *P* value noted<br>*Give P values as exact values whenever suitable.*
☒	☐	For Bayesian analysis, information on the choice of priors and Markov chain Monte Carlo settings
☐	☒	For hierarchical and complex designs, identification of the appropriate level for tests and full reporting of outcomes
☒	☐	Estimates of effect sizes (e.g. Cohen's *d*, Pearson's *r*), indicating how they were calculated

Our web collection on statistics for biologists contains articles on many of the points above.

Software and code

Policy information about availability of computer code

Data collection	No novel software was used
Data analysis	All scRNA-seq analyses were done using R versions 4.1.2 and 4.1.3. We used the Seurat package (4.1.1). MAST version 1.20.0 was used for differential gene expression analysis. We used the singleseq package (version 0.1.2.9000) to run gene set enrichment analysis and compare the enrichment of hallmark pathways between our defined CAF types (Supplementary Data 7).<br><br>Using the lab's analysis pipeline (github.com/BodenmillerGroup/ImcSegmentationPipeline), tiff files were generated from the raw data. Images were produced using histoCAT. The tiff files were used for cellular segmentation based on nuclear and membrane markers using the ilastik software (version 1.3.3). CellProfiler (v3.1.9) was then used to generate cell masks and to calculate mean intensities of each marker per cell. The single-cell data were analysed using R (v4.0.4); raw counts were censored excluding the 99.99 percentile before being arc-sinh transformed using cofactor 1. Graph-based clustering was carried out using the Rphenoannoy clustering algorithm (version 0.1.0). Cells were clustered using FlowSOM (version 2.2.0) clustering as integrated in the CATALYST (version 1.18.1) package. The neighbourhood analysis was carried out analysing the 15 nearest cells in a defined radius of 25 µm of each cells' centroid using imcRtools (version 1.3.7). Differential abundance analysis was carried out using edgeR version 3.36.0 and diffcyt version 1.14.0. Images shown in this study were generated using histoCAT-web and cytomapper (version 1.6.0).

For manuscripts utilizing custom algorithms or software that are central to the research but not yet described in published literature, software must be made available to editors and reviewers. We strongly encourage code deposition in a community repository (e.g. GitHub). See the Nature Portfolio guidelines for submitting code & software for further information.

Data

Policy information about availability of data

All manuscripts must include a data availability statement. This statement should provide the following information, where applicable:

- Accession codes, unique identifiers, or web links for publicly available datasets
- A description of any restrictions on data availability
- For clinical datasets or third party data, please ensure that the statement adheres to our policy

We provide the raw data for all figures as well as the scripts used to generate all figures. IMC RAW data as well as single-cell objects of the IMC data and clustered scRNA-seq data objects have been deposited on zenodo under the following DOI (10.5281/zenodo.5769017 [10.5281/zenodo.5769017] CAF classification with IMC and scRNA-seq). The breast cancer RNA sequencing data are available with accession number E-MTAB-10607 [https://www.ebi.ac.uk/biostudies/arrayexpress/studies/E-MTAB-10607?accession=E-MTAB-10607] on the ArrayExpress database at EMBL-EBI (www.ebi.ac.uk/arrayexpress [www.ebi.ac.uk/arrayexpress]). Publicly available datasets used in this study are available at the Gene Expression Omnibus with the following accession numbers: GSE132465 [https://www.ncbi.nlm.nih.gov//geo/query/acc.cgi?acc=GSE132465] (colon cancer), GSE154778 [https://www.ncbi.nlm.nih.gov//geo/query/acc.cgi?acc=GSE154778] PDAC (small), GSE212966 [https://www.ncbi.nlm.nih.gov//geo/query/acc.cgi?acc=GSE212966] PDAC (big), GSE103322 [https://www.ncbi.nlm.nih.gov//geo/query/acc.cgi?acc= GSE103322] (head and neck squamous cell carcinoma) and in ArrayExpress at EMBL-EBI under the accession numbers E-MTAB-6149 [https://www.ebi.ac.uk/biostudies/arrayexpress/studies/E-MTAB-6149] and E-MTAB-6653 [https://www.ebi.ac.uk/biostudies/arrayexpress/studies?query=E-MTAB-6653] (lung cancer).

Human research participants

Policy information about studies involving human research participants and Sex and Gender in Research.

Reporting on sex and gender	Our dataset contained samples from 14 female breast cancer patients (see Supplementary Data 1). For the publicly available datasets, we did not discriminate between sex.
Population characteristics	Patient data is available in supplementary data 1. Histopathology data was obtained from the respective pathology reports.
Recruitment	All specimens are derived from patients diagnosed with primary breast cancer between 2015 and 2018 at the breast cancer centers at St. Johannes Hospital Dortmund and the Institute of Pathology at Josefshaus (Germany) and the University Hospital Giessen and Marburg (Marburg site, Germany). All specimens were collected in collaboration with the Patients' Tumor Bank of Hope (PATH, Germany).
Ethics oversight	All clinical samples as well as the corresponding clinical information were collected after approval by the Cantonal Ethics Committee Zurich #2016-00215 as well as approval by the ethics committee at Rhenish Friedrich Wilhelms University of Bonn #255/06.

Note that full information on the approval of the study protocol must also be provided in the manuscript.

Field-specific reporting

Please select the one below that is the best fit for your research. If you are not sure, read the appropriate sections before making your selection.

☒ Life sciences ☐ Behavioural & social sciences ☐ Ecological, evolutionary & environmental sciences

For a reference copy of the document with all sections, see nature.com/documents/nr-reporting-summary-flat.pdf

Life sciences study design

All studies must disclose on these points even when the disclosure is negative.

Sample size	We used no statistical methods to pre-determine sample size, since our work was an exploratory study of CAF phenotypes in cancer. We initially defined CAF types using single-cell RNA-Seq data from our previous study (Tietscher, Sandra, et al. "A comprehensive single-cell map of T cell exhaustion-associated immune environments in human breast cancer." Nature Communications 14.1 (2023): 1-20.), performed IMC on the same samples, and validated the resulting classification system using multiple publicly available scRNA-Seq datasets (see Data Availability statement).
Data exclusions	No data was excluded.
Replication	The established fibroblast classification system was verified using publicly available single cell RNA sequencing datasets as well as using imaging mass cytometry on matched samples for the breast cancer cohort. All cell types, clusters and interactions were identified in multiple patient samples and major findings from the scRNA-seq analysis were confirmed on the proteome level by IMC.
Randomization	As this is a descriptive molecular study of fibroblast types in cancer, patient randomisation is not applicable to this study.

Blinding | As this is a descriptive molecular study of fibroblast types in cancer, patient blinding is not applicable to this study.

Reporting for specific materials, systems and methods

We require information from authors about some types of materials, experimental systems and methods used in many studies. Here, indicate whether each material, system or method listed is relevant to your study. If you are not sure if a list item applies to your research, read the appropriate section before selecting a response.

Materials & experimental systems

n/a	Involved in the study
☐	☒ Antibodies
☒	☐ Eukaryotic cell lines
☒	☐ Palaeontology and archaeology
☒	☐ Animals and other organisms
☒	☐ Clinical data
☒	☐ Dual use research of concern

Methods

n/a	Involved in the study
☒	☐ ChIP-seq
☒	☐ Flow cytometry
☒	☐ MRI-based neuroimaging

Antibodies

Antibodies used: All antibodies used in this study are identified in supplementary table 1.

Validation: All antibodies used in this study are validated for their use on FFPE tissues by the manufacturers. All antibodies have been assigned an RRID and can be linked to other publications. Each antibody is further tested in our lab for its tissue specificity in the tissue used, using both fluorescence microscopy and imaging mass cytometry imaging with co-staining of other markers.
